# Therapeutic Hypothermia Ameliorates Apoptosis and Cerebral Injury by Upregulating HECTD1-mediated Ubiquitination and VDAC3 Degradation in a Rat CPR Model

**DOI:** 10.7150/ijms.112837

**Published:** 2026-04-23

**Authors:** Pei Liu, Jiali Wu, Lirong Wang, Jiawei He, Zhanghuan He, Lei Wang, Shen Zhao

**Affiliations:** 1 Department of Critical Care Medicine, Beijing Friendship Hospital, Capital Medical University, Beijing, China.; 2 Department of Critical Care Medicine, Fuzhou University Affiliated Provincial Hospital, Fujian Provincial Hospital, Fujian Provincial Center for Critical Care Medicine, Fuzhou, China.; 3 Fujian Institute of Emergency Research, Fuzhou University Affiliated Provincial Hospital, Fujian Provincial Hospital, Fuzhou, China.; 4 Fujian Provincial Key Laboratory of Emergency Medicine, Fuzhou, China.; 5 Laboratory Animal Center, Beijing Friendship Hospital, Capital Medical University, Beijing, China.

**Keywords:** cardiac arrest, cardiopulmonary resuscitation, therapeutic hypothermia, apoptosis, ubiquitination

## Abstract

**Background and Objective:**

Post-cardiac arrest (CA) brain injury is a leading cause of death and disability in patients with return of spontaneous circulation (ROSC). Our previous study showed that hypothermia-induced VDAC3 ubiquitination attenuated apoptosis of microglia cells following simulated ischemia/reperfusion (I/R). This study aimed to further investigate the role of E3 ubiquitin ligase, HECTD1, in the ubiquitination and degradation of VDAC3, which is involved in hypothermic neuroprotection.

**Methods:**

Sprague-Dawley rats were randomized into two groups, namely the normothermic (T37) group and hypothermic (T33) group, which were further divided into three subgroups: PBS, si-NC, and si-Hectd1. All the rats were subjected to 8 min of asphyxia-induced CA and cardiopulmonary resuscitation (CPR). The core temperature in the T33 and T37 groups were maintained at 33 ± 0.5 °C and 37 ± 0.5 °C, respectively, after 5 min following ROSC and maintained for 6 h. The si-Hectd1 subgroups were administered adeno-associated viral vector with siRNA against rat Hectd1 (si-Hectd1). The PBS and si-NC subgroups were administered the same volume of PBS and nonspecific siRNA, respectively, as controls. Survival time was measured for the resuscitated rats. At 72 h after ROSC, the neurological injuries to rats were evaluated using neurological deficit scores, Serum S100B and neuron-specific enolase levels, hematoxylin and eosin staining, and transmission electron microscopy analysis. Apoptosis-related proteins were measured using western blot assay. The interaction and colocalization of HECTD1 and VDAC3 were evaluated by immunoprecipitation and double immunofluorescence assays, respectively.

**Results:**

Survival time and neurological outcomes, as well as ultrastructural damage, were significantly improved in the T33 group, with the T37 + si-Hectd1 subgroup showing the worst outcome at ROSC 72 h. Compared to the T37 group, the T33 group showed reduced expression of VDAC3, cleaved caspase-3, and BAX but increased expression of HECTD1 and BCL-2 at ROSC 72 h. Additionally, compared to the T37 group, the T33 group showed increased VDAC3 and Hectd1 interaction, resulting in increased VDAC3 ubiquitination at ROSC 72 h; Hectd1 knockdown reversed these effects. The PBS and si-NC subgroups showed no significant differences under both targeted temperatures.

**Conclusions:**

Hypothermia treatment contributed to greater antiapoptotic neuroprotection in the rat CA/CPR model, which may be attributed to the promotion of HECTD1-mediated ubiquitination and degradation of VDAC3.

## Introduction

Hypoxic-ischemic brain injury (HIBI) is a severe sequela directly induced by global ischemia/reperfusion (I/R) following cardiac arrest (CA). HIBI survivors usually progress to brain death or persistent vegetative state after a stable return of spontaneous circulation (ROSC) 1, 2. Current guidelines and recent reviews recommend continuous monitoring of core temperature and active temperature management of post-ROSC patients for a better outcome 3-6. However, the optimal targeted temperature for ROSC patients has not yet been determined. Recent large-scale clinical trials showed no significant positive outcome for hypothermia (33°C) therapy compared to normothermia for patients with ROSC 7, 8. Moreover, the neuroprotective mechanisms of hypothermia are not yet fully understood. Consequently, further preclinical studies are required to clarify the underlying mechanisms of therapeutic hypothermia for patients with ROSC.

Hypoxic-ischemic injury leads to mitochondrial impairment, which results in the release of proapoptotic proteins, followed by cellular apoptosis 9, 10. Voltage-dependent anion channel (VDAC), a porin protein in the outer mitochondrial membrane (OMM), regulates ion and metabolite fluxes, modulates reactive oxygen species production, and is associated with mitochondrial-mediated apoptosis 11, 12. Our previous study demonstrated that therapeutic hypothermia upregulated VDAC3 ubiquitination, thereby protecting murine BV2 microglia from oxygen-glucose deprivation/reoxygenation-induced apoptosis 13. However, the neuroprotective mechanisms of VDAC3 degradation require further investigation.

Hectd1, a member of homologous to the E6-AP C-terminal (HECT) domain-containing E3 ubiquitin ligase family, is widely expressed in both human and murine primary cells, including neuronal cells and fibroblasts 14, 15. Hectd1 may play varying roles in the cerebral ubiquitin-proteasome system based on its target proteins 16, 17. A recent study showed that I/R significantly decreases Hectd1 expression, thereby affecting cell viability and apoptosis 18. Furthermore, bioinformatics analysis revealed that circular RNA (circRNA) *Hectd1* is a promising biomarker for acute ischemic stroke 19. However, studies investigating the effect of hypothermia on Hectd1 expression are limited. Preliminary findings from our proteomics dataset revealed that HECTD1 rose significantly in hypothermia group when compared with normothermia group at 72 h post-ROSC in a rodent cardiac arrest model (see Supplementary Figure S1). In this study, we hypothesized that therapeutic hypothermia may upregulate Hectd1 expression, resulting in VDAC3 degradation. Thus, we investigated the interaction between Hectd1 and VDAC3 using conditional Hectd1 knockdown rats in an experimental model of asphyxial CA and cardiopulmonary resuscitation (CPR).

## Materials and Methods

### Ethics statement

This study was approved by the Animal Welfare and Ethics Committee of Beijing Friendship Hospital, Capital Medical University, Beijing, China (license no. 23-1005). All animals received humane care in compliance with the *Guide for the Care and Use of Laboratory Animals* published by the National Institute of Health 20, 21.

### Animals and grouping

Healthy adult male specific-pathogen free Sprague-Dawley rats (weight: 400-450 g) were purchased from the Experimental Animal Center of Capital Medical University (license number: SYXK 2022-0022). All rats were adaptively reared at 22-25 °C and 60-80% relative humidity and subjected to a 12 h/12 h light-dark cycle for 1 week. Rats were fasted overnight but had ad libitum access to water before the CPR experiment. Animals were randomized into two groups (n = 24/group): normothermia (T37) group and hypothermia (T33) group. The core temperature of the T33 rats was rapidly reduced to 33 ± 0.5 °C using ice packs, an electrical fan, and a cooling blanket at 5 min after ROSC; this temperature was maintained for 6 h using a temperature-controlled blanket (Boruida, Chongqing, China). Subsequently, the T33 rats were rewarmed at a rate of 1.0 °C/h, until their core temperature reached 37 ± 0.5 °C. Furthermore, the core temperature of the T37 rats was maintained at 37 ± 0.5 °C. Both the T37 and T33 rats were then randomly divided into three subgroups (n = 8/group) and administered adeno-associated viral vector (AAV) carrying small interfering RNA (siRNA) against rat Hectd1 (si-Hectd1; si-Hectd1 group), phosphate-buffered saline (PBS; PBS group), or nonspecific negative control (NC) siRNA (si-NC group) via lateral ventricle injection 2 weeks before CPR.

### siRNA preparation and screening

Specific siRNAs were selected to knockdown *Hectd1* expression in the rat brain. Three rat Hectd1 genes, including Hectd1-Rat-265, Hectd1-Rat-2716, and Hectd1-Rat-504, were designed and synthesized as interference targets by Genepharma (Shanghai, China). The specificity of the designed siRNAs was verified by transfecting them into NRK-49F cells using Lipofectamine 2000 (Invitrogen, Carlsbad, CA). The mRNA expression levels of Hectd1 were examined using quantitative polymerase chain reaction (qPCR) (see Supplementary Figure S2). Based on qPCR results, Hectd1-rat-2716-siRNA (5'-GGTGGTAACACTCAGAAAT-3') was selected for the design of si-Hectd1.

### Preparation of recombinant adenovirus particles

Rat Hectd1 (Gene ID: 362736) knockdown was achieved by RNA interference using the AAV-based siRNA approach. The generation of recombinant AAV expressing si-Hectd1 (AAV-GP-7-Hectd1-Rat-2716) and *si-NCs* (AAV-GP-7-NC), as well as viral titer testing, was performed by Genepharma (Shanghai, China). The AAV-293T cells were co-transfected with the RNAi-Mate transfection reagent. After infection, the titers of AAV-GP-7-Hectd1-Rat-2716 and AAV-GP-7-NC were 1.35 × 10^13^ and 2.72 × 10^13^ V.G/ml, respectively.

### Experimental preparation

The animals were anesthetized using an intraperitoneal injection of pentobarbital (45 mg/kg). Orotracheal intubation was completed with a 14 G cannula (Sirui Teaching experiment instrument, Chongqing, China). End-tidal CO2 was continuously monitored with a CO2 analyzer (Capnostream™ 20; Medtronic, CO, USA). Electrocardiogram lead II was recorded. Mean arterial pressure (MAP) and medicine delivery were monitored by inserting a polyethylene catheter (PE-50; Becton-Dickinson, Franklin Lakes, NJ) from the right femoral artery into the descending aorta. Rectal temperature was maintained at 37 ± 0.5 °C using a temperature-controlled blanket. All data were recorded using a four-channel data acquisition system (Taimeng BL-420N, Chengdu, China).

### Establishment of asphyxial CA and CPR

**Figure 1:**
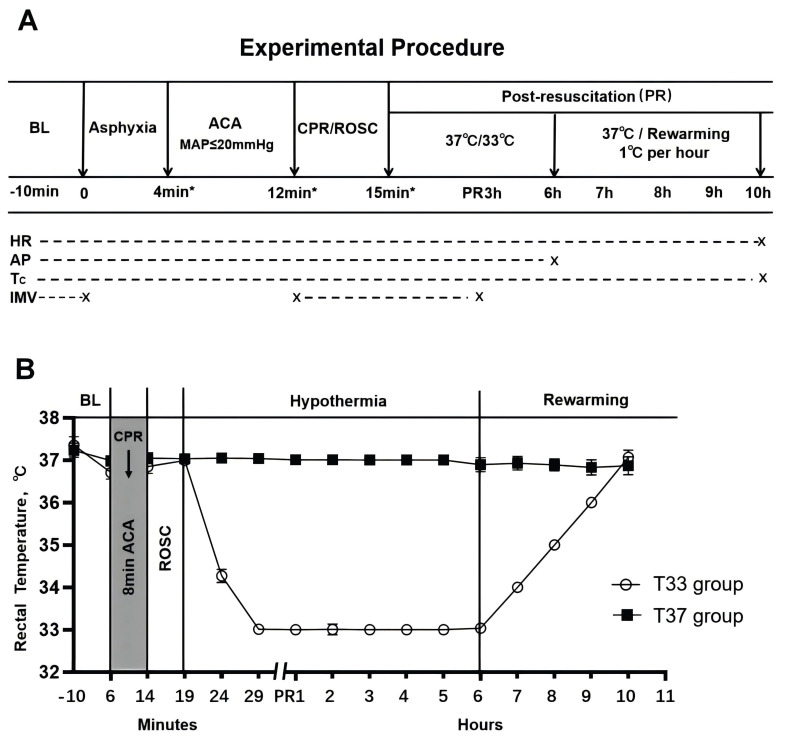
(A) Experimental outline. (B) Core temperature (°C) of the T37 and T33 groups during the experiment. The T37 group includes T37 + PBS, T37 + si-NC, and T37 + si-Hectd1 subgroups, while the T33 group includes T33 + PBS, T33 + si-NC, and T33 + si-Hectd1 subgroups. *Values are expressed as means. BL, baseline; ACA, asphyxial cardiac arrest; CPR, cardiopulmonary resuscitation; ROSC, return of spontaneous circulation; PR, post resuscitation; HR, heart rate; AP, arterial pressure; Tc, core temperature; and IMV, invasive mechanical ventilation.

Baseline measurements were recorded and mechanical ventilation (tidal volume: 0.55 mL/100 g body weight, frequency: 100 breaths/minute, and inspired O2 fraction: 0.21) was initiated 10 min prior to asphyxia induction. Asphyxia was induced by a combination of transarterial rocuronium (1 mg/kg; Merk Sharp & Dohme B.V., Netherlands) administration and endotracheal tube clamping. CA was defined as MAP ≤ 20 mmHg which occurred approximately 4 min following asphyxia. Precordial compression (PC) was initiated 8 min after CA onset to provide a 2:1 compression/ventilation ratio with equal compression and relaxation 22, 23. The compression depth and position were adjusted to maintain a MAP ≥ 20 mmHg. Epinephrine (0.02 mg/kg) was administrated via the femoral artery catheter after 1 min of CPR. In the case of ventricular fibrillation during PC, defibrillation was attempted with a 3-J countershock. ROSC was defined as the return of a supraventricular rhythm with a MAP ≥ 60 mmHg for at least 5 min. If ROSC was not achieved, an additional dose of epinephrine was administered after 3 min of CPR. CPR was halted if ROSC was not achieved after 5 min. Mechanical ventilation with 100% inspired O_2_ was maintained for 1 h following ROSC, and normoxic ventilation was maintained thereafter until the recovery of autonomous respiration. Ventilation weaning and tracheal extubation were performed, followed by sputum suction. The arterial catheter was removed prior to rewarming. Each resuscitated animal was returned to a separate cage, and all the animals were monitored closely for 72 h. The experimental outline and temperature monitoring are summarized in Figure 1.

### Measurements

#### Assessment of neurological function

Neurologic function was evaluated based on the neurological deficit score (NDS) method by two investigators who were blinded to the protocol. NDS, ranging between from 0 (no observed neurologic deficit) to 500 (death or brain death), was recorded at 24-h intervals for 72 h 24.

#### Specimen collection

The animals were euthanized with an overdose of pentobarbital (150 mg/kg, intraperitoneally) after NDS testing at 72 h post resuscitation (PR). Then blood samples were obtained for the measurement of serum biomarkers. For histological evaluation, the animals were sacrificed and subjected to cardiac perfusion with 200 ml of normal saline. Rat brains were obtained and kept on the ice after decapitation. The right frontal cortex of each rat was collected for transmission electron microscopy (TEM) analysis. The right parietal cortex was fixed in 10% formalin. It was then processed through routine dehydration, transparency, embedding, and slicing. Finally, hematoxylin and eosin (HE) staining and immunofluorescence (IF) staining analysis were performed. The left cortex of each rat was quickly separated and stored at -80°C in a refrigerator for CO-immunoprecipitation (CO-IP) and western blot (WB) analyses.

#### Enzyme-linked immunosorbent assay (ELISA)

Blood samples were centrifuged at 1000×*g* for 15 min, and the obtained serum was stored at -80 °C until further analysis. Serum concentrations of neuron-specific enolase (NSE) and S100B were measured using commercially available ELISA kits (E07963r and E08066r, respectively; CUSABIO), according to the manufacturer's instructions. The optical density was determined within 5 min using a microplate reader set to 450 nm.

#### Hematoxylin-eosin (HE) staining

The wax block was sliced into coronal sections (4-μm), dewaxed, debenzolized, and HE stained. Then, the histomorphological changes in the cortex were observed under a microscope. Then HE-stained sections were semi-quantitatively assessed using a histopathological injury scale ranging from 0 to 4^25^.

#### TEM analysis

The tissue sample was rapidly cut into 1 × 1 × 1 mm blocks, fixed in 2.5% glutaraldehyde, and stored at 4 °C. Thereafter, the sample was rinsed with PBS and fixed in 1% osmic acid for 2 h at room temperature. The samples were then dehydrated using an ethanol and acetone gradient and embedded in 812 resin. The embedded tissue blocks were sectioned and observed under a TEM (HT7700, Hitachi) to evaluate the ultrastructural changes in the cortex neurons. TEM images were further Semi-quantitative analysed using Mitochondrial damage index (MDI) ranging from 0 to 3 26.

#### WB analysis

Protein concentrations were measured using a Bradford protein assay kit (Bio-Rad Laboratories). The protein samples (30 µg/lane) were separated by sodium dodecyl sulfate-polyacrylamide gel electrophoresis (SDS-PAGE) and electrotransferred to a polyvinylidene fluoride membrane (Bio-Rad Laboratories). The membrane was blocked with 5% skim milk in Tris-buffered saline containing Tween 20 (TBST) and incubated overnight at 4°C with the following primary antibodies: anti-HECTD1 (sc-517169; Santa Cruz Biotechnology, USA), anti-VDAC3 (55260-1-AP; Proteintech Group, China), anti-BAX (YT0455; ImmunoWay Biotechnology, USA), anti-BCL-2 (YM3041; ImmunoWay Biotechnology), anti-cleaved caspase-3 (CC3; YC0006; ImmunoWay Biotechnology), or β-actin (YT0099; ImmunoWay Biotechnology). The membrane was then washed with TBST and incubated with the corresponding horseradish peroxidase (HRP)-conjugated secondary antibodies, goat anti-mouse IgG or goat anti-rabbit IgG (RS0001 and RS0002, respectively; ImmunoWay Biotechnology). β-Actin was used as an internal control to normalize the relative expression of HECTD1, VDAC3, BAX, BCL-2, and CC3. The optical densities of the protein bands were analyzed using ImageJ software.

#### Double IF staining

Histologic localization of HECTD1 and VDAC3 was determined by IF staining. The brain sections were dewaxed, rehydrated, rinsed with PBS, and incubated with 5% BSA for 2 h at room temperature. Thereafter, the samples were incubated overnight at 4 °C in a wet box with rabbit anti-HECTD1 and anti-VDAC3 polyclonal antibodies. The samples were rewarmed at room temperature for 30 min and then incubated for 2 h with the corresponding fluorescent secondary antibodies at 4 °C in a dark box. Subsequently, the samples were washed thrice with PBS, sealed with an anti-quenching sealing agent containing DAPI, and observed under a fluorescence microscope. Red, green, and blue fluorescence indicated VDAC3, HECTD1, and DAPI expression, respectively. For each slice, five visual fields were randomly captured at 200× magnification using an inverted fluorescence microscope (Leica DMi8, Germany). The fluorescence intensities in each visual field were measured using the Image J software. For the quantification analysis, the captured images were analyzed using the co-localization tools incorporated within the Coloc 2 plugin to obtain a scatter plot. The Pearson colocalization coefficients (PCC) of HECTD1 and VDAC3 were displayed in the colocalized volume, wherein 1, 0, and -1 indicated perfect correlation, no correlation, and perfect inverse correlation, respectively.

#### CO-immunoprecipitation (CO-IP) and immunoblotting (IB) assays

The interaction between HECTD1 and VDAC3 was verified by CO-IP assay. For this, fresh tissue sample (20 mg) was used to prepare 100-200 μL of cortex lysate. The supernatant was collected after ultrasonic homogenization and centrifugation (12000 rpm, 4 °C, and 15 min), and the total protein concentration of the sample was determined using a bicinchoninic acid assay kit (P0010S; Beyotime Biotechnology, China). IP was performed using Co-IP kits (P2179M; Beyotime Biotechnology, China) according to the manufacturer's instructions. Briefly, the protein samples were divided into three parts, which were then treated with 1
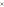
SDS-PAGE loading buffer and boiled for 10 min (positive control; input); incubated overnight with IgG antibodies (negative control); or incubated overnight with rabbit anti-VDAC3 primary antibodies at 4 °C with shaking. The antibody-antigen complexes were mixed with magnetic beads and incubated overnight at 4 °C. After three washes with PBS, the immunoprecipitated proteins were eluted and subjected to SDS-PAGE, followed by immunoblotting with an anti-HECTD1 antibody (Santa Cruz Biotechnology) to detect co-precipitated HECTD1. To further validate this interaction, a reciprocal Co-IP was performed. Cortical lysates were incubated overnight at 4°C with a rabbit anti-HECTD1 antibody. Immune complexes were collected using magnetic beads, washed thoroughly with PBS, and analyzed by immunoblotting with an anti-VDAC3 antibody.

### Statistical analysis

Normal and abnormal distribution data were reported as mean ± standard deviation (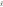
± SD) and median (25% and 75% quartile), respectively. Normal distribution was confirmed with the Kolmogorov-Smirnov test. For normally distributed data, the variables between subgroups were compared using a one-way analysis of variance with Bonferroni's correction. The NDS was compared using the Mann-Whitney *U* test. The survival rates were compared using Fisher's exact test. Survival time was compared using Kaplan-Meier survival analysis. Comparisons of HE staining and TEM findings between the normothermia and hypothermia groups were conducted using the Kruskal-Wallis test. The SPSS 22.0 software (IBM, New York, NY, USA) was used for statistical analysis. *P <* 0.05 indicated statistically significant differences.

## Results

### Characteristics at baseline and during CPR

Of the 136 rats analyzed in this study, 110 were resuscitated successfully. There were no significant differences in the initial ROSC rate, baseline physiological parameters, asphyxia time, PC duration, total epinephrine dosages, defibrillation numbers, and 72 h-survival rates among the six subgroups (all *P* > 0.05) (Table 1).

### Hypothermia improved survival time and neurological outcome in rats following CA/CPR

**Figure 2:**
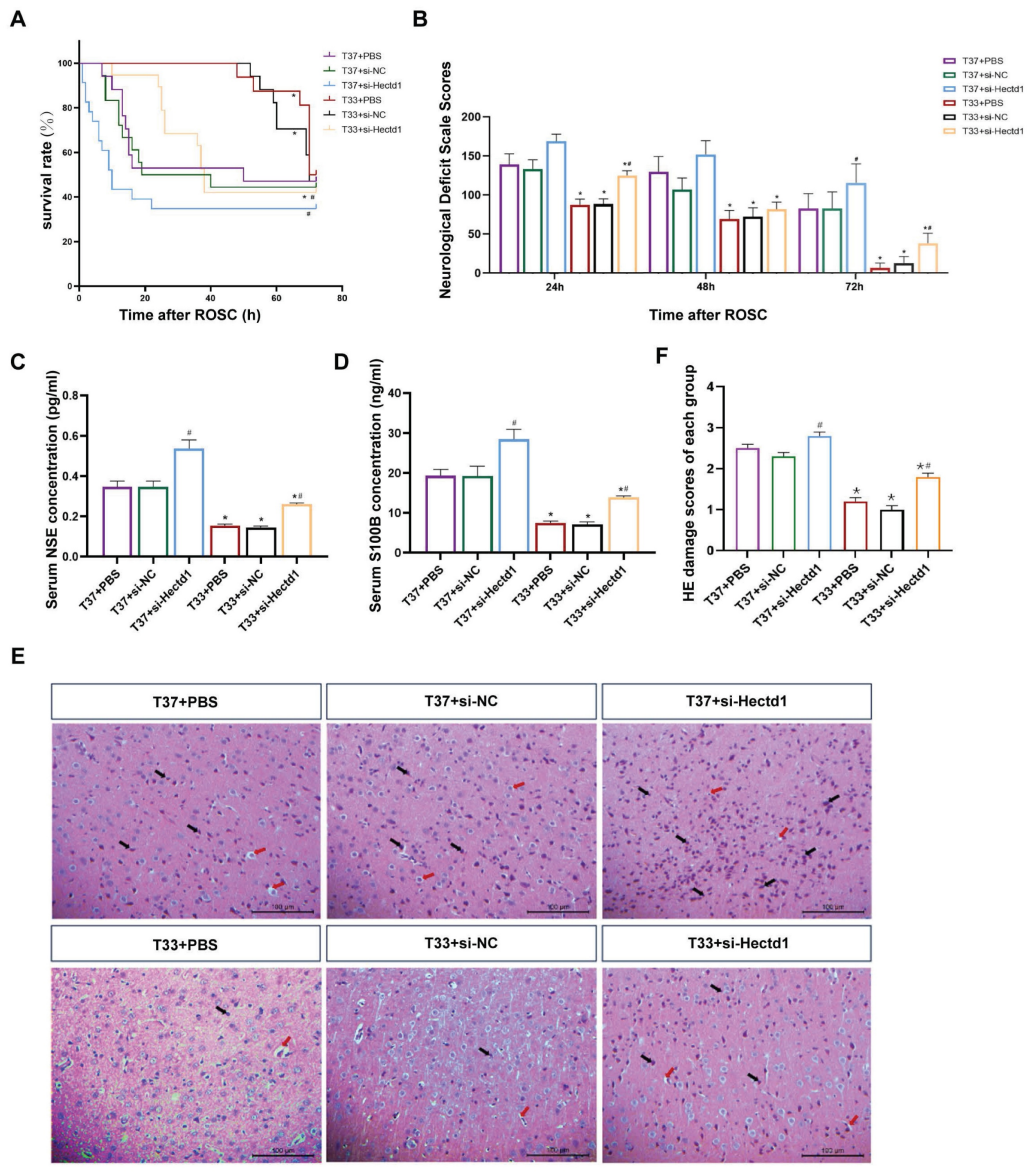
(A) Kaplan-Meier survival curves of T37 + PBS, T37 + si-NC, T37 + si-Hectd1, T33 + PBS, T33 + si-NC, and T33 + si-Hectd1 subgroups. Median survival times of the six subgroups (n = 8), as determined by the Breslow test. (B) NDS of the six subgroups PR (n = 8). (C, D) The serum NSE and S100B levels in the six subgroups (n = 8). (E) Representative HE-stained images (magnification: 200×) of the cerebral cortex samples of the six subgroups (n = 8) at 72-h PR. Black arrows indicate pyknosis, while red arrows indicate vacuolar degeneration. (F) Semi-quantitative analyses for the HE images in the six subgroups (n = 8). ROSC, return of spontaneous circulation; NDS, neurological deficit score; NSE, neuron-specific enolase; HE, hematoxylin and eosin; and PR, post resuscitation. ^*^P < 0.05 vs. T37 group and ^#^*P* < 0.05 vs. PBS or si-NC subgroup.

Despite no significant difference in the 72-h-survival rate, the T33 group demonstrated an overall improved median survival time, NDS, and brain injury biomarkers compared to the T37 group (all *P* < 0.05). Additionally, at both targeted temperatures, si-Hectd1 subgroups exhibited significantly decreased survival time and neurological function compared to the PBS and si-NC subgroups (all *P* < 0.05). Among these, the T37 + si-Hectd1 subgroup showed the worst survival time (*P* = 0.03), NDS (*P* = 0.04), and brain injury biomarkers, including NSE (*P* = 0.02) and S100B (*P* = 0.01) (Figures 2A-D).

Furthermore, HE staining revealed that the cerebral cortex samples of the T37 group showed deeply stained nuclei, pyknotic changes with vacuolated cytoplasm, and interstitial loosening (Figure 2E). However, the T33 group showed significantly improved cell morphology compared to the T37 group (*P <* 0.01). Additionally, our results revealed that at both targeted temperatures, si-Hectd1 subgroups showed significantly aggravated pathomorphologic changes compared to the PBS and si-NC subgroups (all *P* < 0.05) (Figure 2F). Among these, the T37 + si-Hectd1 subgroup showed the worst cerebral pathology (*P* < 0.05 vs. T37 + si-NC group or T37 + PBS). These results indicate that HECTD1 may contribute to hypothermia-induced neurological protection following asphyxial CA and resuscitation.

### Hypothermia induced alterations in the mitochondrial morphology in rats following CA/CPR

**Figure 3:**
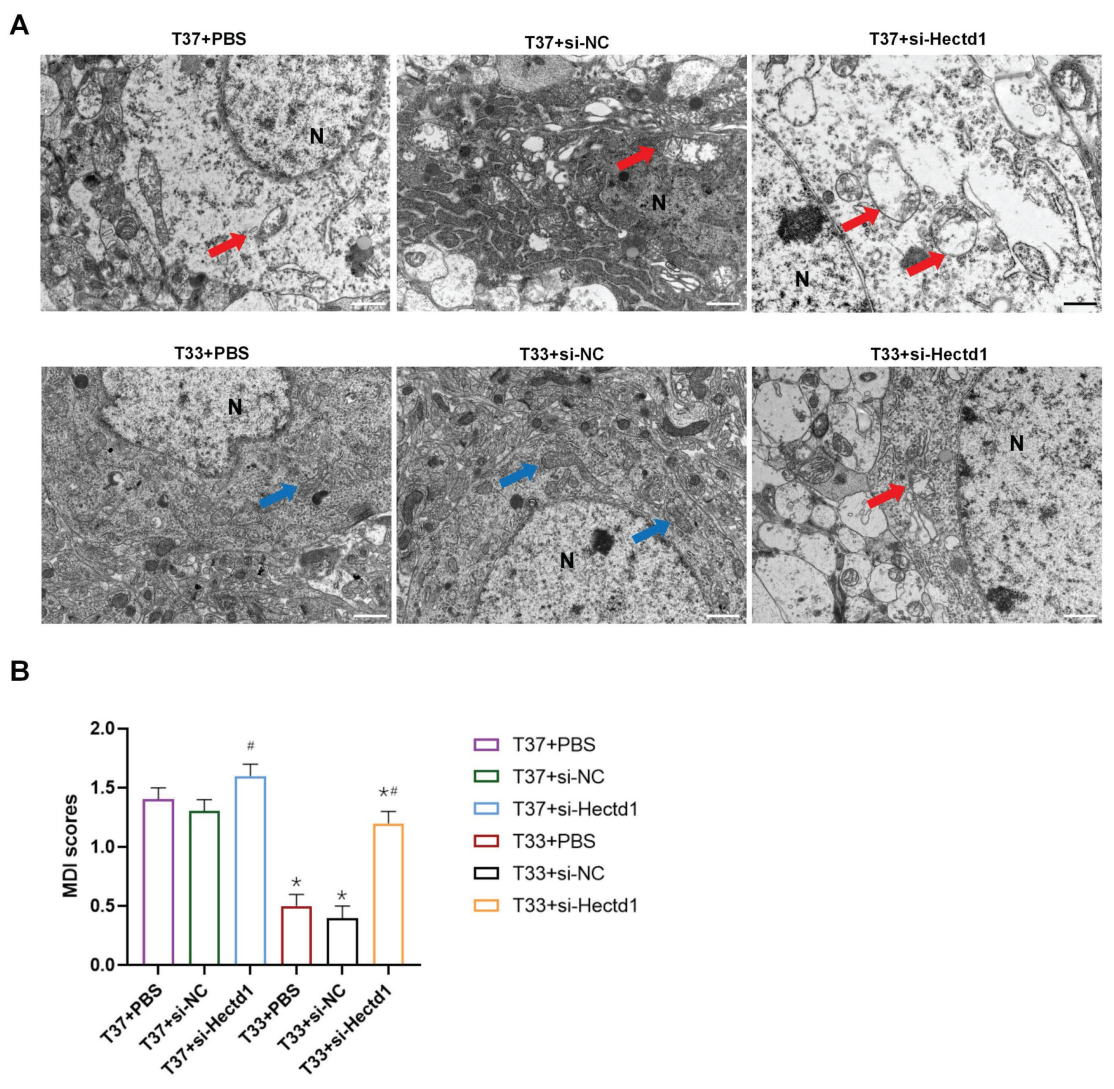
Hypothermia reduced the mitochondrial ultrastructure damage in the cerebral cortex of the CA/CPR rat model. (A) Representative TEM images (Scale bar = 1 μm) of mitochondria of each group. N indicates nucleus, the blue arrow indicates relatively intact mitochondria, and the red arrow indicates swollen mitochondria with fractured cristae. (B) Semi-quantitative analyses for the TEM images in the six subgroups (n = 8). ^*^P < 0.05 vs. T37 group and ^#^*P* < 0.05 vs. PBS or si-NC subgroup.

To demonstrate the effect of hypothermia on mitochondria after CA/CPR, we detected their ultrastructure in the cortex tissues with TEM scanning. TEM images revealed that the T37 group showed severe mitochondrial swelling with disrupted bilayer membrane, vacuolation, and few cristae. However, hypothermia alleviated these morphological alterations in the cortex tissues of the T33 group (*P <* 0.01). The si-Hectd1 subgroups showed significantly altered mitochondrial morphology compared to the PBS and si-NC subgroups at both targeted temperatures (all *P* < 0.01). The T37 + si-Hectd1 subgroup exhibited the most severe mitochondrial damage (*P* < 0.05 vs. T37 + si-NC group or T37 + PBS) (Figure 3). These results indicate that HECTD1 may be involved in the hypothermia-mediated alleviation of alterations in mitochondrial morphology.

### Hypothermia reduced the expression of apoptosis-associated proteins in rats following CA/CPR

**Figure 4:**
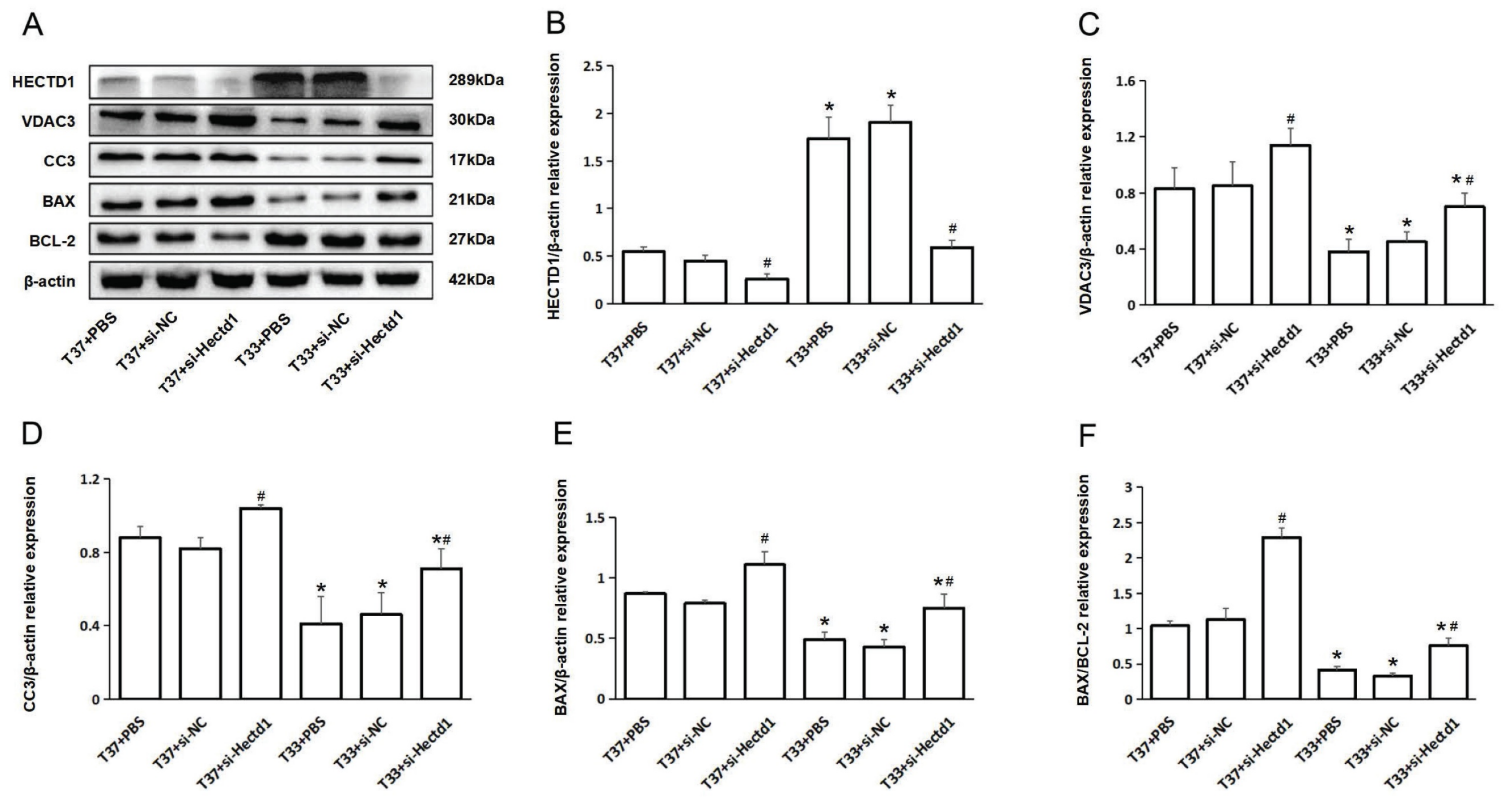
Hypothermia reduced the expression of apoptosis-associated proteins in rats following CA/CPR. (A) WB analysis of HECTD1, VDAC3, CC3, BAX, and BCL-2 expression at 72-h PR (n = 4). (B-F) Quantitative analysis of HECTD1, VDAC3, CC3, BAX, and BCL-2. Values are represented as mean ± SD. ^*^P < 0.05 vs. T37 group and *^#^P* < 0.05 vs. PBS or si-NC subgroup.

To further analyze the effect of hypothermia on the expression of apoptosis-associated proteins, we conducted WB analysis of HECTD1, VDAC3, CC3, BAX, and Bcl-2. Compared to the T37 group, the T33 group showed significantly increased HECTD1 expression (*P* < 0.05). In contrast, the T33 group showed significantly reduced VDAC3, CC3, and BAX expression as well as BAX/BCL-2 ratio compared to the T37 group (all *P* < 0.05), consistent with the results of our previous study. However, the si-Hectd1 subgroups showed significantly increased levels of the aboving markers at both target temperatures compared to the PBS and si-NC subgroups (all *P* < 0.05). And the T37 + si-Hectd1 subgroup exhibited the highest level of pro-apoptosis-associated protein expression (*P* < 0.05 vs. T37 + si-NC group or T37 + PBS) (Figure 4). These results suggest that hypothermia may increase HECTD1 expression, resulting in the promotion of VDAC3 degradation in the PR period.

### Hypothermia enhanced Hectd1 and VDAC3 interaction in rats following CA/CPR

**Figure 5:**
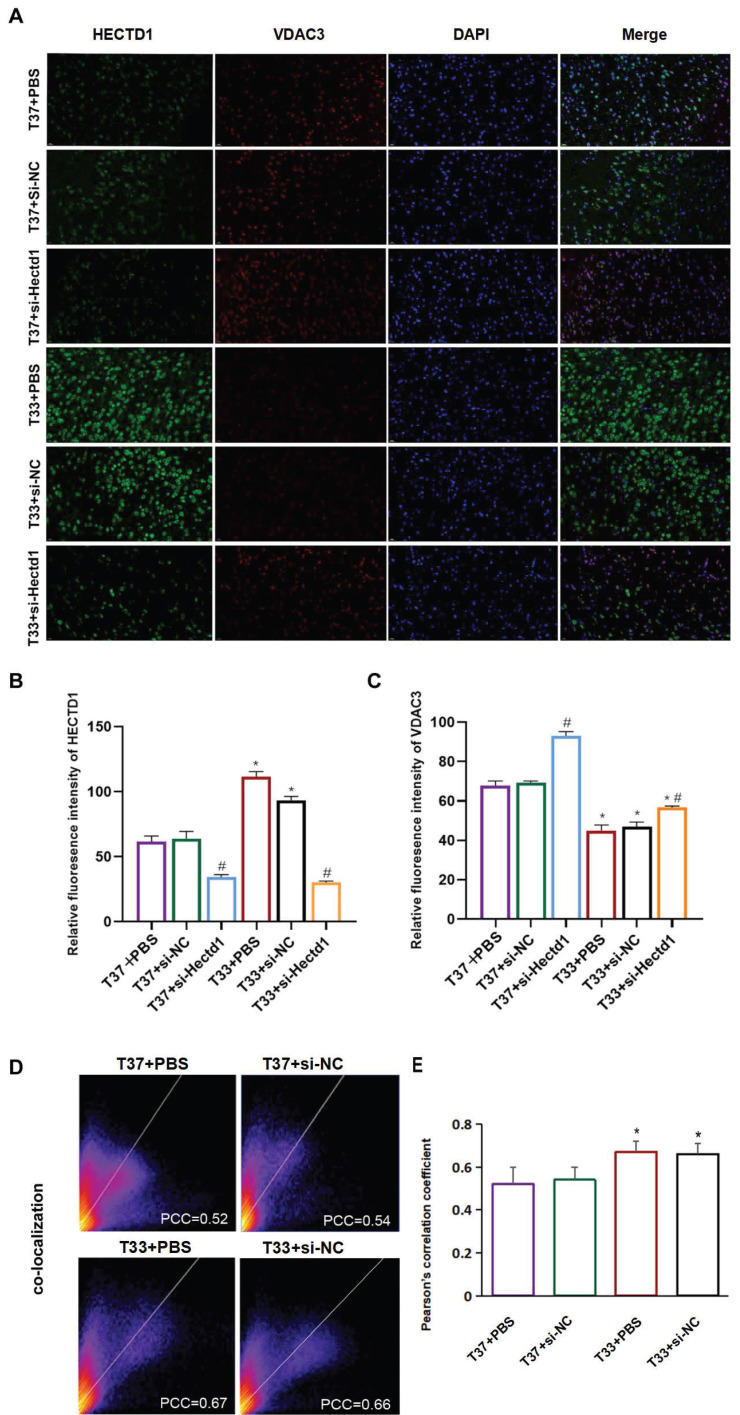
Hypothermia enhanced Hectd1 and VDAC3 co-localization in rats following CA/CPR. (A) IF images (magnification: 200×) showing the density and distribution of neurons labeled with anti-HECTD1 (green) and anti-VDAC3 (red) antibodies. Blue fluorescence indicates DAPI-labeled nuclei. (B, C) Statistical analysis of HECTD1 or VDAC levels between T33 and T37 groups. (D) Pixel distribution scatter plot: yellow pixels along the diagonal represent colocalized intensity; PCC, Pearson's correlation coefficient. (E) PCC values were analyzed for at least 3 different fields for each group. Values are represented as the mean ± SD (n = 8). ^*^P < 0.05 vs. T37 group.

The results of double IF staining using anti-HECTD1 (green) and anti-VDAC3 (red) antibodies revealed that VDAC3 (an OMM marker) was colocalized with HECTD1 in the cerebral cortex of rats following resuscitation (Figure 5A). The fluorescence intensities of both HECTD1 and VDAC3 were consistent with the trends observed with the IB assay (Figures 5B, C). As indicated in Figure 5 D, the distribution scatter plot showed a representative image derived from the PCC obtained using the Coloc 2 plugin. Compared to the T37 group, the T33 group showed a significantly increased fluorescence intensity of HECTD1, resulting in a higher PCC (*P* < 0.01) (Figure 5 E). Interestingly, fluorescence intensity of VDAC3 was significantly higher in the T37 + si-Hectd1 group than in the T33 + si-Hectd1 group, suggesting that Hectd1 may partly interpret the decrease in VDAC3 expression (*P* < 0.01) (Figures 5A, C). These results indicate that hypothermia may enhance HECTD1 expression and its colocalization with VDAC3.

**Figure 6:**
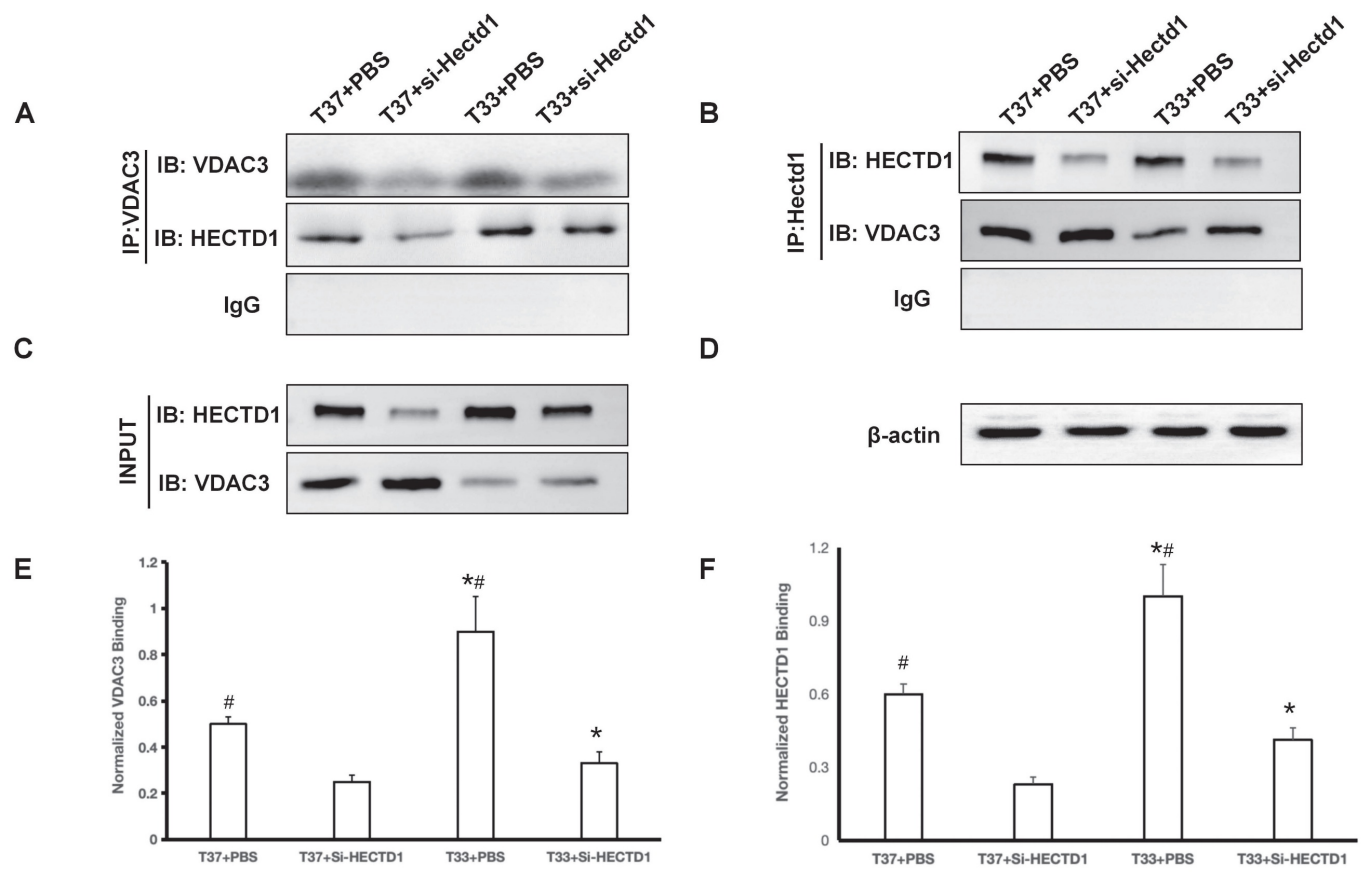
Hypothermia enhanced HECTD1 interacted with VDAC3 in rats following CA/CPR. (A-B) Co-IP assays demonstrate reciprocal binding in the PBS and si-Hectd1 subgroups of T37 and T33 groups. Lysates were immunoprecipitated with antibody against HECTD1 (IB: VDAC3) and VDAC3 (IB: HECTD1) as shown. (C) Input controls of VDAC3 and HECTD1. (D) Loading control β-actin in the lysates. (E-F) Quantitative analysis of protein interaction. The relative binding affinity between HECTD1 and VDAC3 was quantified using the formula: (VDAC3 intensity in Co-IP / HECTD1 intensity in Co-IP) ÷ (VDAC3 intensity in Input / Loading control intensity in Input) and (HECTD1 intensity in Co-IP / VDAC3 intensity in Co-IP) ÷ (HECTD1 intensity in Input / Loading control intensity in Input). Data are presented as mean ± SEM from four independent experiments. Values are presented as the mean ± SD. ^*^P < 0.05 vs. T37 group and *^#^P* < 0.05 vs. si-Hectd1 subgroup.

Co-immunoprecipitation assays confirmed a direct interaction between HECTD1 and VDAC3. Lysate from cortex tissues was immunoprecipitated with an anti-HECTD1 antibody. Subsequent IB assay with an anti-VDAC3 antibody revealed a clear band specifically in the HECTD1 immunoprecipitate, but not in the control IgG precipitate (*P* < 0.01 vs. si-Hectd1 group) (Figure 6A). Reciprocal co-IP using an anti-VDAC3 antibody for pull-down also successfully co-precipitated HECTD1, further validating this interaction (*P* < 0.01 vs. si-Hectd1 group) (Figure 6B). Hypothermia treatment led to a marked upregulation of HECTD1 expression, as shown in the input lysates (*P* < 0.01 vs. T 37 group) (Figure 6C). This also reflects a change in binding affinity by normalizing the co-IP signal to the respective input levels (Figure 6E-F). The results suggest that exposure to hypothermia treatment upregulated HECTD1 expression and potentiated its interaction with VDAC3, thereby resulting in enhanced HECTD1-mediated ubiquitination of VDAC3.

## Discussion

This study demonstrated that mild hypothermia following ROSC alleviated neurological dysfunction and apoptosis in the CA/CPR rat model. Additionally, this study revealed that the beneficial effect of hypothermia was associated with VDAC3 ubiquitination *in vivo*, consistent with our previous findings 13. Furthermore, this study indicated that, compared to the normothermic treatment, hypothermic treatment increased the interaction between the E3 ubiquitin ligase HECTD1 and VDAC3, which retained the improved neurologic outcomes and survival time in the CA/CPR rat model.

VDACs are considered highly conserved proteins of the OMM and are known to be involved in many cellular processes, including regulation of energy metabolism/ion transport and mitochondria-mediated apoptosis, autophagy, or ferroptosis, by interacting with cytosolic and mitochondrial proteins 27-29. Many studies have shown that mitochondrial permeability transition pore formation, dependent on VDACs and other component proteins, induces apoptosis during I/R 30, 31. Electrophysiological and immunocytochemical observations showed an increase in VDAC expression or activation in neurons during apoptosis and/or oxidative damage 32, 33. Furthermore, studies have shown that post-translational modifications of VDACs, including phosphorylation, helix docking, or ubiquitination, affect neuronal function 34-36. Increasing evidence indicates that the stability, aggregation, and physiological properties of VDACs are highly dependent on the temperature conditions 37, 38. However, only a few studies have been conducted on the association between hypothermia-induced VDAC ubiquitination and organ protection during I/R. In this study, we demonstrated that the increase in the level of VDAC3, a temperature-sensitive protein, is associated with neurological dysfunction during PR. However, VDAC3 homeostasis induced by therapeutic hypothermia contributed to the attenuation of mitochondria-mediated apoptosis and neuroprotection in the CA/CPR rat model.

Ankyrin repeat (AR) domains can identify and interact with a variety of intracellular substrates, which serve a critical role in biological processes, including the ubiquitination signaling pathway 39. HECTD1 is a key AR-containing E3 ubiquitin ligase, and it regulates neuroinflammation and neuron cell apoptosis by ubiquitinating its target proteins 14, 40. Furthermore, the downregulation of HECTD1 expression is associated with stress-induced apoptosis and endothelial injury during I/R 16, 18, 41. Several studies found that circRNA Hectd1, derived from the Hectd1 gene, is a reliable biomarker for acute ischemic stroke 42, 43; However, the downstream pathways of circRNA Hectd1-mediated regulation of neurological deficit development have not been fully explored. Further studies should explore the protein-protein interaction networks associated with HECTD1 to determine its target proteins for ubiquitination. In this study, we found that hypothermia enhanced HECTD1 expression and promoted its interaction with VDAC3 during PR. Therefore, our results revealed that VDAC3 could serve as a substrate for HECTD1-mediated ubiquitination, contributing to mitochondrial apoptosis in the CA/CPR rat model.

We observed that the T37 + si-Hectd1 subgroup showed higher VDAC3 expression than the T33 + si-Hectd1 subgroup. This explains the complex regulation of VDAC3 expression, including HECTD1-mediated VDAC3 degradation under hypothermic conditions. Additionally, this result indicates the efficiency of siRNA delivery by AAV. *Hectd1* knockout using transgenic techniques, such as clustered regularly interspaced short palindromic repeats/Cas9, may enable more efficient gene targeting to produce desired phenotypes in rats 44.

In this study, there was no statistical difference in NDS between the subgroups of the T37 group within 48 h. This finding may be attributed to severe myocardial dysfunction induced by normothermia during the early post-resuscitation period. As post-resuscitation myocardial function subsequently improved, the elevated expression of HECTD1 demonstrated more pronounced neuroprotective effects by 72 hours post-resuscitation. Accordingly, the 72-hour timepoint after ROSC was selected for the current investigation, as it aligns with critical outcomes including neurological function, survival, and definitive histological evidence of cellular injury. Future studies examining the temporal dynamics of HECTD1 and VDAC3 expression may further elucidate the neuroprotective mechanisms underlying therapeutic hypothermia.

This study has some limitations. First, we used polyclonal antibodies against VDAC3 for the CO-IP experiment, which could not effectively prevent persistent nonspecific interactions between the antibody complex and HECTD1. This limitation could affect the comprehensiveness of our mechanistic interpretation, though our controlled experimental conditions maintain internal validity. Second, the cellular origin of the observed HECTD1 and VDAC3 expression changes remains partially unresolved. Without co-localization studies using specific markers for neurons and microglia, neuroprotective process cannot be exclusively attributed to a particular cell type. This represents an important avenue for future investigation. Finally, while the animal protocol used in this study was well controlled, the application in clinical practice might be limited due to the unavailability of target protein detection methods. The technical limitation, combined with the current lack of validated clinical-grade assays for real-time protein quantification, necessitate cautious interpretation of these preclinical results until further methodological validation studies are conducted. Nevertheless, we observed the protein expression patterns following the CA/CPR and targeted temperature management protocols, which could provide insights into the mechanisms underlying the neuroprotective benefits of therapeutic hypothermia.

## Conclusion

Our study demonstrated that therapeutic hypothermia improved neurological dysfunction and survival outcomes in a rat model of asphyxial CA and resuscitation. This may be attributed to increased Hectd1-induced VDAC3 degradation, which results in the attenuation of mitochondria-mediated apoptosis of neurons.

## Supplementary Material

Supplementary figures.

## Figures and Tables

**Figure 7:**
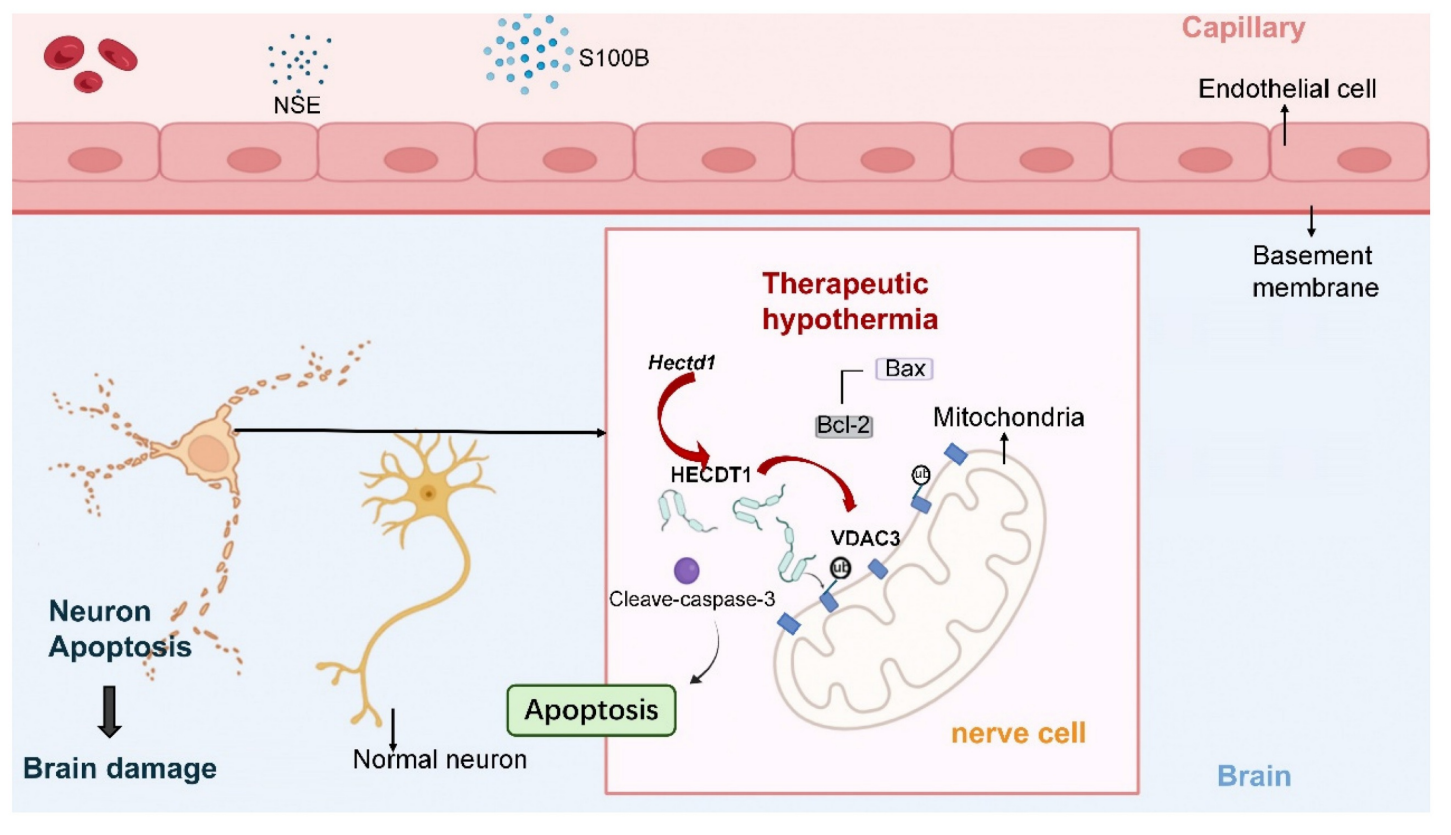
Proposed mechanism underlying the neuroprotective effect of therapeutic hypothermia after cardiac arrest. Therapeutic hypothermia (TH) increases the expression of the E3 ubiquitin ligase HECTD1, which promotes ubiquitination-dependent degradation of the mitochondrial channel protein VDAC3. The reduction of VDAC3 is associated with decreased mitochondrial apoptotic signaling and lower levels of cleaved caspase-3, ultimately attenuating neuronal apoptosis and improving neurological outcomes.

**Table 1:** Baseline and outcomes of resuscitation

Variables	T37+PBS	T37+si-NC	T37+si-Hectd1	T33+PBS	T33+si-NC	T33+si-Hectd1	*P* value
Body weight, g	431±15	433±13	440±10	435±12	428±15	430±16	0.92
Rectal temperature, ℃	37.1±0.4	37.0±0.2	37.3±0.5	37.0±0.3	37.0±0.6	37.1±0.5	0.80
ETCO2, mmHg	51±4	53±3	52±5	51±4	53±5	52±4	0.87
Heart rate, bpm	451±39	457±29	442±24	456±25	449±27	463±21	0.74
MAP, mmHg	129±6	129±7	130±7	133±8	134±9	135±11	0.52
Asphyxia time, s	252±28	244±17	238±17	236±11	238±21	238±29	0.68
PC time, s	172±75	184±69	162±68	148±58	187±63	187±77	0.82
Total epinephrine dosages, ml	0.15±0.1	0.13±0.2	0.14±0.1	0.15±0.1	0.13±0.1	0.15±0.1	0.93
Defibrillation numbers	0.6±0.7	0.5±0.5	0.5±0.5	0.6±0.7	0.5±0.5	0.6±0.5	1.00
ROSC/total CPR	17/21	18/22	23/28	16/20	17/21	19/24	1.00
72-hour survival/ROSC	8/17	8/18	8/23	8/16	8/17	8/19	0.95

Note: Values are expressed as means ± SD. ETCO2, End-tidal carbon dioxide; MAP, Mean aortic pressure; Asphyxia time, time from start of asphyxia to MAP≤20mmHg; PC, precordial compression; ROSC, return of spontaneous circulation; CPR, cardiopulmonary resuscitation

## Abbreviations

CA: cardiac arrest
ROSC: return of spontaneous circulation
I/R: ischemia/reperfusion
T37: normothermic
T33: hypothermic
CPR: cardiopulmonary resuscitation
si-Hectd1: siRNA against rat Hectd1
HIBI: hypoxic-ischemic brain injury
VDAC: voltage-dependent anion channel
OMM: outer mitochondrial membrane
HECT: homologous to the E6-AP C-terminal
circRNA: circular RNA
AAV: adeno-associated viral vector
siRNA: small interfering RNA
PBS: phosphate-buffered saline
NC: negative control
qPCR: quantitative polymerase chain reaction
PC: precordial compression
NDS: neurological deficit score
PR: post resuscitation
TEM: transmission electron microscopy
HE: hematoxylin and eosin
IF: immunofluorescence
WB: western blot
IP: immunoprecipitation
ELISA: enzyme-linked immunosorbent assay
NSE: neuron-specific enolase
HE: hematoxylin-eosin
SDS-PAGE: sodium dodecyl sulfate-polyacrylamide gel electrophoresis
TBST: Tris-buffered saline containing Tween 20
PCC: Pearson colocalization coefficients
IB: immunoblotting
