# Supplementary material for: Therapeutic Hypothermia Ameliorates Apoptosis and Cerebral Injury by Upregulating HECTD1-mediated Ubiquitination and VDAC3 Degradation in a Rat CPR Model
Source: Int J Med Sci. 2026 Apr 23;23(6):1992–2005. doi: 10.7150/ijms.112837 (PMC13181373; doi:10.7150/ijms.112837)
Supplement: Supplementary file 1 — Supplementary figures. [file ijmsv23p1992s1.pdf]

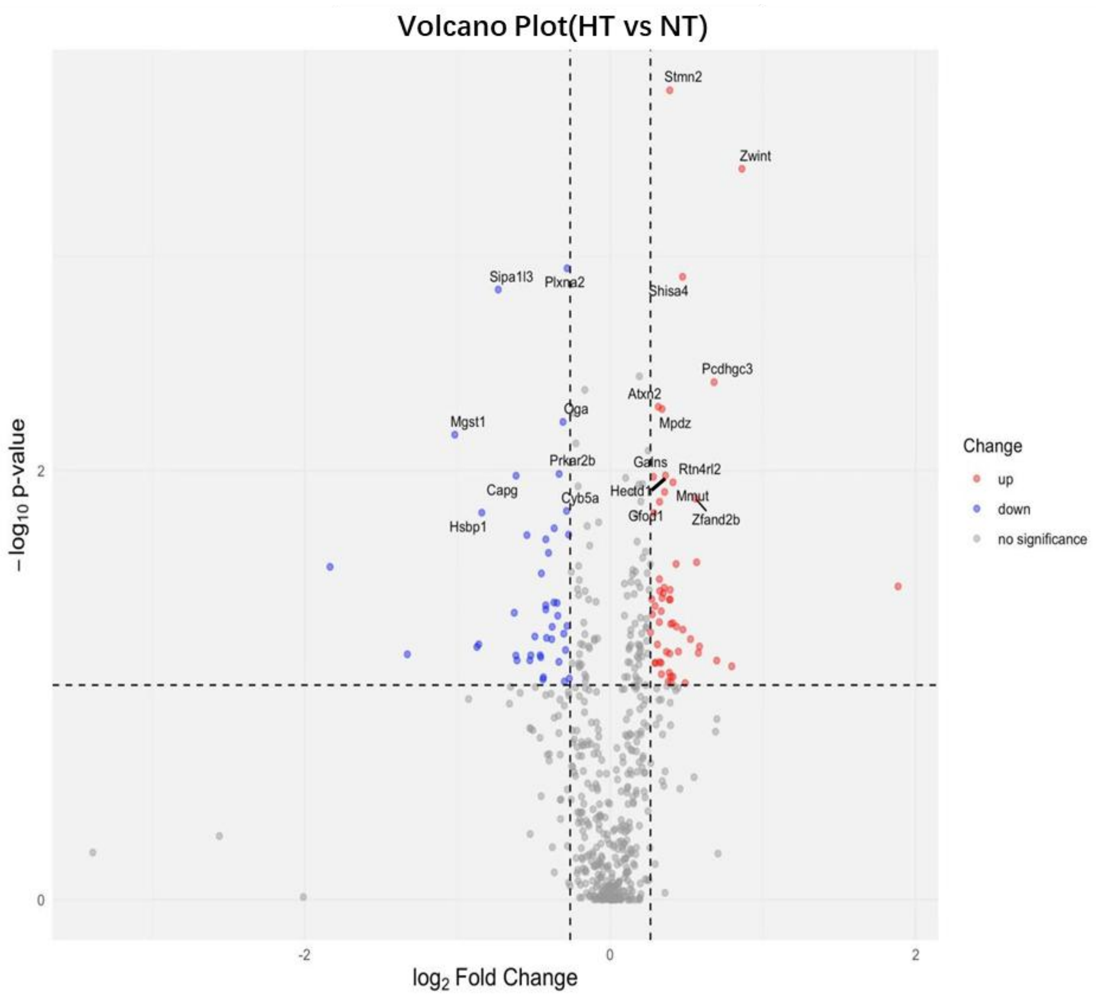

Figure S1. Proteomic analysis revealed that Hectd1 expression was significantly upregulated in the hypothermia group compared to the normothermia group. (Protein screening criteria for hypothermia group vs normothermia group: Adjusted P-value (Tukey's method) < 0.1, Fold change (FC) > 1.2 or < 0.83. A total of 105 proteins are shown, including red - upregulated proteins: 57 with $P < 0.1$ and FC > 1.2; blue - downregulated proteins: 48 with $P < 0.1$ and FC < 0.83.

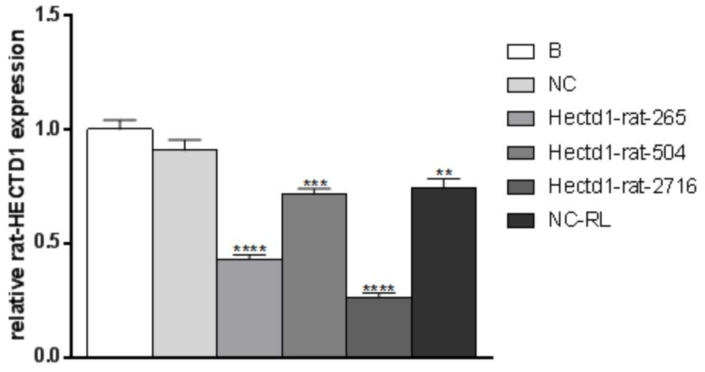

Figure S2. The qPCR experiment used rat-ACTB（Actin Beta） as the internal reference to calibrate the expression levels of rat-HECTD-1 gene at 48 hours. Hectd1-rat-2716-siRNA significantly downregulated the expression of the rat HECTD-1 gene compared to the other two siRNAs. B: Blank; NC: Negative Control; NC-RL: Negative Control-Random Library.
